# Exposure Levels of Adult Patients during Radiographic Examinations: Sinuses and Coastal Grill Cases at the Ngaoundere Regional Hospital, Cameroon

**DOI:** 10.1155/2019/5452149

**Published:** 2019-04-30

**Authors:** Guiswe Gnowe, Fouda H. P. Ekobena, Amvene J. Mbo, Guena M. Neossi

**Affiliations:** ^1^Department of Biomedical Sciences, Faculty of Sciences, University of Ngaoundere, Cameroon; ^2^Higher Institute of Sciences, Health Technics and Management of Garoua, Cameroon; ^3^University Institute of Technology, University of Ngaoundere, Cameroon; ^4^Faculty of Medicine and Biomedical Sciences of Garoua, Cameroon; ^5^Department of Radiology and Medical Imaging, Regional Hospital of Ngaoundere, Cameroon

## Abstract

**Background:**

The purpose of this study was to estimate the doses delivered to adult patients during explorations of the sinuses and coastal grill whose attention in dosimetric terms is neglected because of the low demand for diagnosis. But yet dosimetric values are very high.

**Materials and Methods:**

The present study was transversely descriptive and was conducted between April and July 2016. The data were collected on 50 adult patients of mass of 70±10kg at the Regional Hospital of Ngaoundere (HRN). The dose at the entrance of the patients' skin was evaluated through the theoretical methods derived from the Davies model according to the 75th percentile calculations.

**Results and Discussion:**

The entrance skin doses obtained in mGy were, respectively, of 7.2±0.2 for sinuses and 5.27±0.1 for coastal grill. The present study found many variations in doses during radiological investigations. These variations allowed us to understand that the notions of quality of the radiographic image, insurance, and quality control of the radiological equipment are tributary, abstract, and often theoretical because doses delivered are not sufficiently optimized.

**Conclusion:**

The dosimetric analysis remains very worrying because the various procedures show that an improvement of the practices especially with respect to the technical parameters and the protocols must be considered. Therefore a strengthening of radiological protection skills of radiological manipulators (continuing education and retraining) would contribute to a better protection of patients.

## 1. Introduction

In radiology, patient dosimetry is a good match between image quality and the low dose process. Continuous evaluation by means of periodic inspections and the fight to reduce the doses delivered should be at the center of the activities (after the structure in charge through effective monitoring in the field and in all structures equipped with X-ray tubes). In fact, the doses delivered to patients during medical imaging examinations are not sufficiently controlled and the working protocols for a given examination differ from one hospital service to another. These variations indicate that good imaging technique is needed to reduce the dose to patients at the lowest practicable level to respond to the clinic and make the diagnosis [1]. These large variations in the doses delivered to patients for the same examination also show a crucial importance for the study of dose variations [2], for quality assurance and standardization of protocols [3], in order to guarantee the optimization of doses to patients. We must give ourselves the means to measure and to evaluate this energy and to verify that patients are not overexposed during standard radiography.

Patient dosimetry is a functional operation parameter such as high voltage or kilovolt (kV), current intensity or milliamperage (mAs), morphotype, posteroanterior (PA), anteroposterior (AP), focus-skin distance (FSD), and field and filtration [4]. It should be noted that the diagnosis is dependent on the quality of the radiological image and therefore of the dose at the entrance surface of the skin. In radiology, the dosimetric quantities selected for the Diagnostic Reference Levels (DRLs) determinations are the input dose (ESD in mGy) and the Dose-Area Product (DAP in Gy.cm^2^) [5].

The entrance skin dose (ESD) in conventional radiography can be obtained by calculation from mathematical methods or measured by a thermoluminescent dosimeter (TLD) [6]. These methods have relatively small differences. The calculation or mathematical method appears reliable and is an effective alternative for measuring the entrance skin dose [7]. The determination of patient doses is an essential part of the optimization process, such as the adequacy of the image and radiation in radiological examination. The optimization of doses delivered during the radiological examinations, by the determination of the DRLs, the control of the quality of the installations, and radiological procedure, makes it possible to minimize the risk related to these irradiations by reducing the dose received by the patient. The concept of DRL is specific to medical exposures and should not be confused with that of “individual dose limit” which is applied in these areas of radiation protection of workers and the public. Reference levels are tools for improving practices and optimizing doses. These levels exist only for routine explorations in radiological examination. Respect for the reference levels is not, by itself, a criterion of good practice. The primary goal, inseparable from dosimetry, is the diagnostic quality of low dose images [5].

Our objectives were the following:

- Determine the sociodemographic profiles of the patients received for these particular examinations.
- Determine the dose levels delivered to adult patients based on radiological exploration by theoretical models of dose calculation.
- Compare the results obtained with those obtained elsewhere according to our realities.

## 2. Materials and Methods

The study was monocentric, descriptive, and transversal. It took place between April and July 2016 (four months), in the Radiology and Medical Imaging Department of the Ngaoundéré Regional Hospital. This department is equipped with a GENERAL ELECTRIC X-ray tube, model 5192454, whose maximum voltage was around 150 kV.

### 2.1. Target

Included in this study were all adult subjects irrespective of sex and age who had undergone a blondeau radiographic examination and coastal grill during the period. The data collected include age, sex, and anthropometric data of patients with weights between 60 and 80 kg.

### 2.2. Collection and Statistical Analysis

The information was collected using a sheet adapted according to the model of the Institute of Radiation Protection and Nuclear Safety (IRNS) dosimetric evaluation. The irradiation parameters used (kV, mAs), the geometric parameters (FFD, FSD), and the anthropometric data (age, sex, and weight) were processed and analyzed with the strictest anonymity. The calculations, the output of the tube, and the entrance skin dose (ESD) patients were donned by Sphinx Plus^2^ V.5.1.0.6 and Excel 2010.

### 2.3. Calculation of the Dose

The irradiation and geometric parameters involved in the calculation of the dose at the entrance of the skin were calculated (by means of 75^th^ percentiles, standard deviations). The calculation of the dose at the entrance of the skin began by calculating the output (output) of the X-ray tube according to the model of [8]:

(1) OPmR=A×6,53×10−4mRmAskV2−1×kV2×mAs

where A is a constant equal to 0.5; 0.8 and 1 are for single-phase, three-phase, and high frequency tubes generators. As part of our study, the X-ray tube was three phases. The yields obtained in (mR) were converted to mGy, mAs^−1^ by multiplication at a factor of 0.00877/mAs [9].

Then, the calculation of the dose at the entrance of the skin proper for each patient was according to the Davies model [10]. This is thanks to the values of irradiation parameters and geometric and output values. We chose this model because it integrates all the elements directly, involved in the realization of radiographic examinations and the definition of the dose at the entrance of the skin [11]. The logic of the arrangement of radiographic parameters in this formula obeys well to that realized during the obtaining of the radiographic images.

(2) ESDmGy=OP×kV802×mAs×100FSD2×BSF

Only images of good qualities accepted for interpretation were considered. The study was authorized by the ethics committee of the hospital structure.

## 3. Results

The results obtained only were presented in Table 4.

### 3.1. Sociodemographic Data

For 50 subjects, men were more represented than women (66%), with a sex ratio (H/F=1.94).

Regarding the male sex: the mean age was 40.33 and the standard deviation was 8.54 years; the range of age was 26-48 years for sinuses.

The mean age was 27.2 and the standard deviation was 6.64 years; the range of age was 20-38 years for coastal grill.

With regard to the female sex: the mean age of the women was 27.2 and the standard deviation was 6.64 years; the range of age was between 20 and 38 years for sinuses. The mean age was 33.16 and the standard deviation was 6.22 years; the range of age was between 32 and 41 years for coastal grill.

### 3.2. Yield of X-Ray Tube

The different values of the output are essential for the process of optimizing the dose delivered and the quality of the images. They are directly a function of the high voltage (kV) and the load (mAs). For sinuses, in mGy. (mAs)^−1^, the output ranged from 0.19 to 0.29 in PA. For coastal grill, in mGy (mAs)^−1^, output ranged from 0.19 to 0.25 in AP.

### 3.3. Overall Knowledge of the Irradiation Parameters Used during Exams

The parameters presented in this study are those that made it possible to obtain good quality according to the different morphotypes of the patients. These parameters are very important in the process of optimizing the dose delivered and improving the quality of the images. After calculation (mGy), the third quartile was 7.02 and standard deviation was 0.2 for sinuses. The minimum and maximum entrance skin dose ranged from 5.8 to 12.2 in PA. The third quartile was 5.27 and standard deviation was 0.1 for coastal grill. The minimum and maximum entrance skin dose skin ranged from 4.98 to 7.67 in AP.

### 3.4. Study of Comparative Values

The values obtained (mGy) were compared with those obtained elsewhere by the same approaches. The entrance skin dose was 7.02±0.2 for sinuses in PA. The entrance skin dose was 5.27±0.1 for coastal grill in AP.

## 4. Discussion

The population of our study consisted of patients admitted to the Radiology Department of the Regional Hospital of Ngaoundere. The study included 50 adult patients of more than 20 years, both sexes, with a weight between 60 and 80 kg. X-ray examinations were performed by manipulators of different rank and seniority. The parameters used for the same examination were variable according to the habits of each manipulator. Many manipulators do not have the same rules of good practice. These different parameters were observable on the quality of the snapshots and the disparity of the input doses delivered to the patients. The results obtained were calculated using the 75^th^ percentile method. The good practicing in radiographic involves a permanent adaptation of the technical procedure related to the equipment, the choice of parameters (irradiation and geometry), and possible accessories, which constantly influence the dose received by the patient. The resumption of radiographic examinations is an important preventable or at least reducible factor of overexposure of patients. Effective remediation and reduction measures such as periodic assessment and dosimetric testing must be considered.

### 4.1. Perception of Sociodemographic Data

Table 1 presents the sociodemographic data of the patients. Ages, sex, and weight of the patient are the most important parameters in considering the interpretation of radiological images. The technical choices of performing an examination are also a function of the morphotype of the patient. Therefore, some clinicians would ignore the importance of these parameters for imaging, coupled with the fact that some patients in our environment are not always able to accurately age [12]. Much more according to [13], the level of awareness of clinicians in Cameroon is very low and superficial, which affects the quality of their imaging demands. Despite the compliance of certain test reports, it should be noted that contact with the patient during imaging examinations should allow us to complete the missing parameters.

### 4.2. Importance of X-Ray Tube Performance

Table 2 presents the estimation of the X-ray tube yielding from the theoretical model proposed by [8]. It is important to consider tube efficiencies for each specific run to control tube power and to take corrective action if deviations from normal are noted. Obtaining a quality image is the primary function of the efficiency of the X-ray tube. The power of the tube becomes an optimization factor in the evaluation of the “dead” performance of the X-ray tube. The devices are calibrated beforehand by the manufacturers and the parameters applied on the console for any examination are not the same at the exit of the tube.

### 4.3. State of Exposure and Context

Table 3 presents the following:

(i) The result of the exploration of the sinuses was in the order of 7.02-0.2 mGy. This result was greater than 3.2 mGy estimated by [14], but similar to 5.06 mGy, estimated by [15]. Mali and Cameroon share some common points of delay in the radiation protection of patients.

(ii) At the end of the exploration of the coastal grill, we obtained 5.27±0.1mGy. This result appears very high to our knowledge because the thoracic framework is a bone structure. It can be explored from low settings. According to [16], the reduction in voltage and charge (mAs) has been shown to be effective in reducing the dose delivered by 40% without impairing the diagnostic quality of images, especially for bone structures.

### 4.4. Knowledge of Radiation Protection during Standard Radiological Examinations

Table 4 presents a comparative statement between our results and others elsewhere. This table indicates that the values are obtained by [14, 15]. These differences were specifically associated with exceeding nominal values as proposed by the manufacturer despite the absence of protocols. This study is devoted to examinations that are weakly “realized” and whose evaluation in dosimetric terms is often not considered. Yet, they are strongly radiating. We note that these results cannot be extrapolated to all radiology departments in Cameroon because the study was based on the principle of voluntary. Nevertheless, these values are indicatives from the dosimetric point of view. Despite the existence of a law governing radiation protection and an agency in charge of radiation protection, the absence of technical protocols in the examination room makes it difficult to control the doses delivered to patients. As observed elsewhere, the absence of texts in favor of radioprotection and or extracts in this radiology department proves the embryonic state of radiation protection of patients in this department. In practice, it is possible to avoid unnecessary radiation despite attendance routine trainees and make good quality pictures. But this observation is still very alarming, when X-rays are carried out daily by some caregivers who have no background profile of the field but rather converted into radiology manipulators. The latter then have no idea or then a rough knowledge of the texts in favor of radiation protection. All these observations point to [17]; unlike developed countries, in the sub-Saharan African countries, particularly in Cameroon, legislative and regulatory frameworks are either nonexistent or are implemented in an approximate manner and the practices of radiation protection of patients are poorly documented in a context of expanding medical imaging. If the report is real, it is necessary to note the embryonic and precarious state of the standards and devices in favor of the radioprotection of the patients. Moreover, the lack of qualified personnel in radiation protection and the lack of resources and continuing training in radiological protection should only lead to approximate visions of radiation protection if the manipulators were recycled; endowing more than the manipulation console offers a platform for adjustment irradiation parameters and therefore dosimetric controls. As imaging spreads to the most remote areas of the country, there is an urgent need to optimize work protocols. This optimization of the protocols could be achieved by continuous training; the display in the examination rooms of the working protocols and a permanent comparison of the values to the references and permanent correction measures could partly reduce the observed radiology differences.

## 5. Conclusion

The risk of irradiation is potentiated by the nonobservance of the basic principles of patient protection. Far from trivializing the exposure of patients to the ionizing radiation, we must instead be vigilant and educate staff about it and we are proposing the measures to reduce the skin entrance doses. However, the dosimetric analysis remains very worrying. The analysis of the different procedures shows that an improvement of the practices especially with respect to the technical parameters and the protocols, combined with a reinforcement of the radioprotection competences of the radiology manipulators (continuous training and recycling), will contribute to a better radioprotection of the patients. The creation of a regulatory framework allowing not only the radiation protection of the patients but also the personnel must be effective on the ground, by means of the periodic controls and the evaluations of the professional practices. This work is also an essential call to work in the mastery of technical and standardization of examination protocols to control and optimize the doses delivered to patients for a better match of the image quality and the low dose over the area.

## Figures and Tables

**Table 1:** Sociodemographic characteristics.

Radiography	Sex	Amount	Age (years)	Weight (kg)
Sinuses	M	14	26-48 40.33±8.54	65-78 71.33±5.77
	F	11	26-54 33.57±9.04	61-78 69.42±5.02
Coastal grill	M	19	20-38 27.2±6.64	63-78 68.5±3.62
	F	6	32-41 33.16±6.22	65-76 70.33±4.52

M: male, F: female.

**Table 2:** Output of X-ray tube in mR  and mGy.  (mAs)^−1^.

Radiography	Projection	Output (mR)		Output mGy. (mAs)^−1^	
		Min	Max	Min	Max
Sinuses	PA	22.07	33.43	0.19	0.29
Coastal grill	AP	22.07	29.38	0.19	0.25

PA: posteroanterior, AP: anteroposterior, Min: minimum, max: maximum.

**Table 3:** Technical parameters used.

Radiography	Projection	m	mAs	DFF	FSD	ESD		3^rd^ quartile	SD
						Min	Max		
Sinuses	PA	65-80 66.1±1.5	32-50 41.9±2.4	1.3-1.5 1.2±0.04	1.0-1.3 1.1±0.04	5.8	12.2	7.02	0.2
Coastal grill	AP	65-75 66.7±2.0	40-55 43.2±2.1	1.5-1.8 1.6±0.07	1.2-1.5 1.3±0.06	4.98	7.67	5.27	0.1

kV: kilovolt, mAs: milliampere second, FFD: focus-film distance, FSD: focus-skin distance, ESD: entrance skin dose, SD: standard deviation.

**Table 4:** Comparison between our values and some of them.

Radiography	Projection	Our study	DRL	Mali	Saudi Arabia
			Europe	[15]	[14]
Sinuses	PA	7.02±0.2	-	5.06	3.2
Coastal grill	AP	5.27±0,1	-	-	-

## Data Availability

The data used to support the findings of this study are available from the corresponding author upon request.
